# Effect of timing of umbilical cord clamping on maternal and neonatal outcomes: A protocol for systematic review and network meta-analysis

**DOI:** 10.1097/MD.0000000000015283

**Published:** 2019-04-19

**Authors:** Liping Yu, Yue Sun, Yi Shang, Min Yin

**Affiliations:** a Department of Nursing, Rehabilitation Center Hospital of Gansu Province; b School of Nursing, Lanzhou University, Lanzhou City; c Department of General Surgery, The Second Hospital of Lanzhou University; d Department of Nursing, The First Hospital of Lanzhou University, Gansu Province, China.

**Keywords:** infant, network meta-analysis, systematic review, umbilical cord clamping

## Abstract

**Background:**

Umbilical cord clamping is one of the most commonly used medical or complementary medical interventions. The different timing of cord clamping may have any significant impact on public health. However, the results remain controversial. The aim of the study was to evaluate and compare the effect of different timing of umbilical cord clamping on maternal and neonatal outcomes.

**Methods:**

A systematic literature search for relevant articles will be conducted in the Cochrane Central Register of Controlled Trials, PubMed, Embase, and Chinese Biomedical Literature Database from their inception to December 2018. Any randomized controlled trial (RCT), case-control study, observational study, that reported the effect of different timing of cord clamping will be included regardless of sample size. There are no language restrictions. Mortality and risk of iron-deficiency anemia will be used to assess the clinical effect. Risk of bias assessment of the included RCTs will be conducted by the Cochrane risk of bias tool and the Newcastle–Ottawa Scale is used to assess observational studies. All statistical analyses will be performed using Stata V.15.0. A modified version of Grades of Recommendation, Assessment, Development, and Evaluation will be used to assess the quality of evidence in network meta-analysis (NMA).

**Results:**

The results will be published in a peer-reviewed journal.

**Conclusion:**

This will be the first NMA to evaluate and compare the effect of different timing of umbilical cord clamping. We hope that the results of this NMA will help clinicians and caregivers make more appropriate choices when clamping umbilical cord.

## 1 Introduction

Since the mid-18th century, the timing of umbilical cord clamping has been, and still is, focus of controversy.^[1]^ Umbilical cord clamping is one of the most commonly used medical or complementary medical interventions. The different timing of cord clamping may have any significant impact on public health. Over the past decade, refurbishment of this topic has led to the development of several clinical and physiological research.

Delayed umbilical cord clamping may increase the volume of blood transferred from placenta to infant and improve outcome in preterm infants by allowing timing for physiologic transition.^[2]^ Previously early clamping was normal practice in preterm infants, reflecting concerns about harm from delayed resuscitation, hypothermia, jaundice, and polycythemia.^[3–5]^ As we all know, clinical practice guidelines (CPGs) are one way of providing information on evidence-based and are trustworthy. However, the timing of umbilical cord recommended by the CPGs from different organizations is different. The American College of Obstetricians and Gynecologists recommended that the timing of umbilical cord clamping of term infants and premature infants is delayed by 30 to 60 seconds.^[6]^ It was believed by the American Heart Association that for newborns who do not need resuscitation, delay the umbilical cord for at least 1 minute.^[7]^ European Consensus Guidelines on the Management of Neonatal Respiratory Distress Syndrome in Preterm Infants suggested if the condition of the newborn is stable, the umbilical cord should be delayed for at least 60 seconds after delivery to promote placenta transfusion to the fetus.^[8]^ It was emphasized that clamping the umbilical cord after the umbilical cord stops, it can prevent new anemia in children by International Confederation of Midwives and International Federation of Gynecology and Obstetrics.^[9]^

Network meta-analysis (NMA) is popularly used to evaluate different interventions. It has the advantage of allowing indirect comparisons of multiple interventions for estimation and ranking their orderings even though direct head-to-head comparison studies are lacking.^[10–11]^ The value of NMAs for health-care decision making has been recognized and accepted by different health technology assessment and funding agencies worldwide.^[12–14]^

Our study will evaluate the effect of different timing of umbilical cord clamping, and will provide valuable information to help policy to choose the safest timing of umbilical cord clamping.

## 2 Methods

### 2.1 Eligibility criteria

#### 2.1.1 Type of study

Any randomized controlled trials (RCT), case-control study, observational study that reported the effect of different timing of cord clamping will be included regardless of sample size. There are no language restrictions.

#### 2.1.2 Type of patients

New infants of any gestation.

#### 2.1.3 Type of interventions

We will include studies that reported the effect of different timing of cord clamping; trials with cord milking are not eligible.

#### 2.1.4 Type of outcomes

Primary outcomes

1. Mortality;
2. Risk of iron-deficiency anemia.

Secondary outcomes

1. Incidence of jaundice;
2. Blood transfusion, incidence of postpartum hemorrhage;
3. Major morbidity, including necrotizing enterocolitis, chronic lung disease, retinopathy of premature, hyperbilirubinemia, intraventricular hemorrhage.

### 2.2 Data source

This will include electronic searches of the Cochrane Central Register of Controlled Trials, PubMed, Embase, and Chinese Biomedical Literature Database; at the same time, the reference lists of published reviews and retrieved articles will be checked for additional trials.

Search strategy of PubMed was as follows:

#1 “Umbilical Cord” [Mesh]
#2 Umbilical Cord[Title/Abstract] OR Umbilical-Cord[Title/Abstract] OR umbilicus[Title/Abstract] OR Umbilical Cords[Title/Abstract]
#3 #1 OR #2
#4 clamp∗[Title/Abstract]
#5 #3 AND #4
#6 Best Match Filters: Humans

### 2.3 Study selection

We will use EndNote X7 to manage citations from databases. Two independent reviewers will check the title and abstract of each citation retrieved according to eligibility criteria. The full texts of potentially relevant studies will be retrieved for further assessment. Disagreements will be resolved by discussion or consultation of a third author. Two trained reviewers will independently extract the required data from the included studies for inclusion by using Microsoft Excel 2013 (Microsoft Corp, Redmond, WA, www.microsoft.com), and another trained reviewer will check the extracted data. Data will be extracted from eligible studies including publication details, general characteristics of include trials (name of first author, year of publication, number of centers, setting, total sample size), detail of participants (gender, age, country), and intervention characteristic as well as outcomes. Any missing data will be acquired by contacting by email or telephone. When this is not possible, we will conduct the analysis using available data.

### 2.4 Risk of bias of individual studies

We will use different quality assessment tools to assess different types of studies.

Two trained authors will assess the methodological quality of RCT by using the Cochrane risk of bias assessment (Cochrane Handbook for Systematic Review Interventions),^[15]^ which was composed of 6 domains: random sequence generation, allocation concealment, blinding of all participants, including patients, personnel and outcome assessors, incomplete outcome data, selective reporting, and other source of bias. We will evaluate methodological quality as low, high or unclear risk of bias. Disagreements will be resolved by a third investigator.

The Newcastle–Ottawa Scale is a risk of bias assessment tool for observational studies that is recommended by the Cochrane Collaboration.^[15–16]^ The tool comprises 8 items which are representativeness of the exposed cohort, selection of nonexposed cohort, ascertainment of exposure, demonstration that outcome of interest was not present at start of study, comparability of cohorts on the basis of the design or analysis controlled for confounders, assessment of outcome, was follow-up long enough for outcomes to occur, and adequacy of follow-up of cohorts.^[16]^

### 2.5 Statistical analysis

#### 2.5.1 Pairwise meta-analysis

We will perform pairwise meta-analyses of direct evidence using Stata V.15.0. Dichotomous data will be expressed as odds ratios with 95% confidence intervals and continuous outcomes will be presented as mean differences with 95% confidence intervals.^[17]^ We will present 95% confidence intervals for all outcomes. The *χ*^2^ test was used to analyze heterogeneity. If the *P* value ≥.1 and *I*^2^ ≤50%, it suggests that there is no statistical heterogeneity, we will use the Mantel Haenszel fixed effects model for meta-analysis. If the *P* value <.1 and *I*^2^ >50%, we consider that there is heterogeneity in the study. We will use a random effects model and conduct sensitivity analysis and subgroup analysis to detect the source of heterogeneity.

#### 2.5.2 Network meta-analysis

A Bayesian mixed treatment comparison approach will be performed by WinBUGS 14^[18]^ and to determine the comparative effectiveness of treatments.

In this method, noninformative prior distributions and Markov chain Monte Carlo simulations will be used, and 4 parallel chains will be applied, with at least 5000 or more iterations (as needed) to derive the corresponding 95% credible intervals.^[2]^ We will compared eviance and deviance information criterion statistics in fitted consistency and inconsistency models, and examine inconsistency plots to evaluate inconsistency using the Brooks–Gelman–Rubin diagnostic (ie, a conflict between direct and indirect evidence).^[19]^ Surface under the cumulative ranking area will be used to rank the different timing of umbilical cord clamping, a larger surface under the cumulative ranking means a more effective intervention.^[20]^ Comparison-adjusted funnel plots will be used to assess the effects of the sample size on the results. All the figures will be generated using Stata 15.0 software.

Begg and Egger funnel plot method through StataV.15.0 will be performed to help distinguish asymmetry due to publication bias when applicable.^[21–22]^

### 2.6 Quality of evidence

A modified version of Grades of Recommendation, Assessment, Development, and Evaluation (GRADE)^[24–26]^ will be used to assess the quality of evidence in NMA. For confidence in specific pairwise effect and treatment rankings estimated in the NMA the following domains will be considered (study limitations, consistency of effect, imprecision, indirectness, and publication bias). The GRADE system specifies 4 levels of quality of evidence:

1. High quality for randomized trials; or double-upgraded observational studies;
2. Moderate quality for downgraded randomized trials; or upgraded observational studies;
3. Low quality for double-downgraded randomized trials; or observational studies; and
4. Very low quality for triple-downgraded randomized trials; or downgraded observational studies; or case series/case reports.

Two researchers will independently be in charge of evaluation for each included study. The GRADE process will be completed using the CINeMA software, which is developed by the Cochrane Statistics Methods Group for evaluating confidence in the results of NMA.^[23–24]^

## 3 Conclusion

To the best of our knowledge, there are no NMA comparing the clinical effect of different timing of umbilical cord, although a few studies have assessed the effect of timing of umbilical cord clamping.

Therefore, the objective of this systematic review and NMA will summarize the direct and indirect evidence to assess effect of different timing of umbilical cord. We hope that the results of this NMA will help clinicians and caregivers make more appropriate choices when clamping umbilical cord.

## Author contributions

LP and YS planned and designed the study. YS and MY tested the feasibility of the study. YS and MY provided methodological advice, considered for ideas and overall structure of the article and revised the manuscript. LP and YS wrote the manuscript. All authors approved the final version of the manuscript.

**Methodology:** Liping Yu, Yue Sun, Yi Shang, Min Yin.

**Supervision:** Liping Yu, Yue Sun, Yi Shang, Min Yin.

**Writing – original draft:** Liping Yu, Yue Sun, Yi Shang, Min Yin.

**Writing – review and editing:** Liping Yu, Yue Sun, Yi Shang, Min Yin.

Min Yin orcid: 0000-0002-3974-2090.
